# Charge-state Resolved Infrared Multiple Photon Dissociation (IRMPD) Spectroscopy of Ubiquitin Ions in the Gas Phase

**DOI:** 10.1038/s41598-017-16831-2

**Published:** 2017-11-29

**Authors:** Yijie Yang, Guanhua Liao, Xianglei Kong

**Affiliations:** 1 State Key Laboratory and Institute of Elemento-Organic Chemistry, College of Chemistry, Nankai University, Tianjin, 300071 China; 2 Collaborative Innovation Center of Chemical Science and Engineering, Nankai University, Tianjin, 300071 China

## Abstract

In this study, we obtained for the first time the direct infrared multiple photon dissociation (IRMPD) spectra of ubiquitin ions in the range 2700–3750 cm^−1^. Ubiquitin ions with different charge states showed absorption in the two regions of 2940–3000 cm^−1^ and 3280–3400 cm^−1^. The increase of the charge state of ubiquitin ions broadened the absorption peak on the high-frequency side in the second region, indicating some hydrogen bonds were weakened due to Coulomb interaction. It is also found that the relative intensity of the absorption peak in the first region compared to the absorption peak in the second region increased with increasing charge state, making the IRMPD spectra charge-state resolved. Although it is usually reasonable to suggest the origin of the absorption in the range 2940–3000 cm^−1^ as the C-H bond stretching modes, the results show significantly reduced absorption after the deuteration of all labile hydrogen atoms. A possible explanation for this is that the coupling coefficients between the C-H vibrational mode and other selective modes decreased greatly after the deuteration, reducing the rate of energy redistribution and probability of consecutive IR absorption.

## Introduction

Protein ions in the gas phase have been the subject of extensive investigation since the introduction of electrospray ionization (ESI)^1^. In order to gain better understanding of the structures and kinetics of these gaseous species, several techniques based on mass spectrometry (MS) have been developed, including collisionally activated dissociation (CAD)^2,3^, electron-capture dissociation (ECD)^4–8^, H/D exchange^6,9–11^, and ion mobility measurement^11–15^. Among these methods, infrared multiple photon dissociation (IRMPD) spectroscopy, or infrared photodissociation (IRPD) spectroscopy, has been carried out to obtain the spectroscopic signatures of these biomolecules^6,16–38^.

In combination with theoretical calculations, IRMPD spectroscopy has been successfully applied to the structural analysis of amino acids and short peptides^16–30^. However, the method is difficult to apply to intact proteins directly, as the stability of these large ions could inhibit their fragmentation and render the photon-induced action untraceable^30,31^. In addition, large molecules readily isomerise in the gas phase and this causes changes in their IR spectra, modifying the absorption cross-section at the pump wavelength. To our knowledge, the largest ion studied using direct IRMPD spectroscopy to date is the Trp-cage, which consists of 20 amino acids^30^. In order to obtain useful IRMPD spectra of large protein ions, two strategies have been previously investigated. The first of these involves combination of IR absorption with other activation methods. With the help of electron-capture activation prior to IR irradiation, Oh *et al*. reported the first IRPD spectra of ubiquitin (Ubi) ions in the range 3050–3750 cm^−1^ ^6^. IR–UV double resonance spectroscopy is also a powerful tool for measurement of vibrational spectra of polyatomic molecules. With this method, Nagornova *et al*. obtained the spectrum of cytochrome c (12+) recently^32^. In their experiment, cold ions in an ion trap were irradiated by a tunable optical parametric oscillator (OPO) laser and a UV laser in turn, then UV-induced photofragmented ions were recorded at each IR wavenumber. The second strategy involves the collection of IRPD spectra of protein ions by employing small ions or molecules as messengers. These messengers would link to the protein ions through non-covalent interactions, and thus could be readily removed by IR activation. For example, Oomens *et al*. studied potassiated cytochrome c ions by using the free electron laser for IR experiments (FELIX) and obtained their spectra in the amide I and II spectral regions^33^. Wang *et al*. collected the IR spectra of multiply charged holomyoglobin ions (hMb^n+^) by monitoring loss of the heme moiety^34^.

In comparison with IR absorption techniques, the IR-messenger approach is easier to perform, although the isolation of the unstable non-covalent complexes can be challenging. The main issue associated with the use of messengers is that the effects of their binding on the conformation of the gaseous protein ions are not easy to ascertain in most cases. Recently, a new method that detected the ions generated by laser induced ejection from the ion-doped helium droplets was applied to record the infrared spectra of ubiquitin and cytochrome c by von Helden’s group^35^. Interestingly, they found that for proteins ions with high charge states, a new band appeared at 1480 cm^−1^, which was red-shifted from the amide II band at 1550 cm^−1^ observed for ions with low charge states. Combined with theoretical study, the results were interpreted in terms of Coulomb-driven transitions in secondary structures^35^. In this paper, we show that direct IRMPD spectroscopy can be successfully performed for intact protein ions of ubiquitin (76 mer), when a high-power (~600 mW) OPO IR laser is used. Remarkably, our results show that IRMPD spectra of different charged ions of the same protein can be resolved.

## Results and Discussion

**Figure 1:**
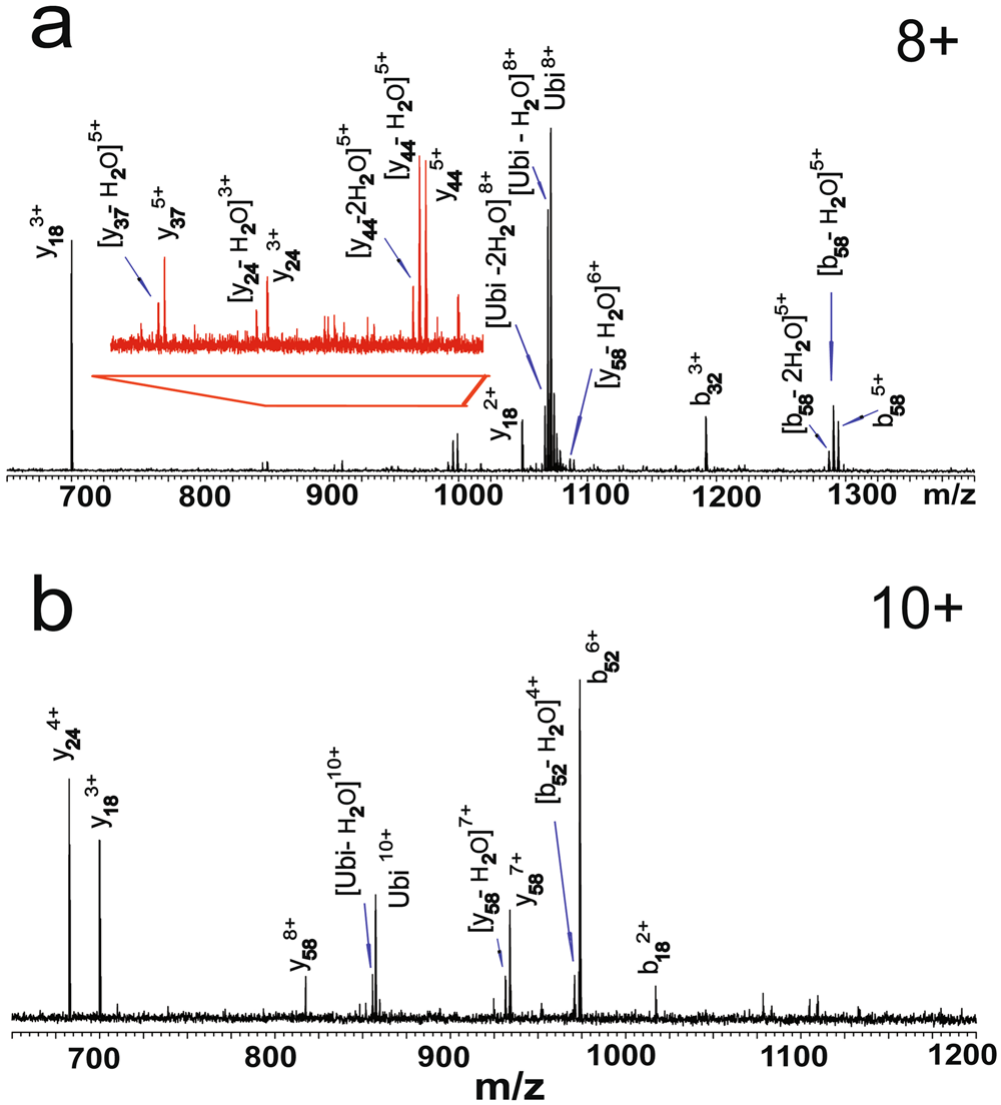
IRMPD mass spectra of (**a**) [Ubi+8 H]^8+^ and (**b**) [Ubi+10 H]^10+^. Spectra were obtained by IR irradiation at 3370 cm^−1^ for 2 s and 4 s, respectively.

Ubiquitin ions with different charge states were generated by ESI, and then isolated and trapped in the cell of the FT ICR mass spectrometer. Owing to the high energy of the IR laser, direct IR-induced fragment ions could be detected. For example, the strong absorption at 3370 cm^−1^ caused direct dissociation of ubiquitin ions with different charges using a short irradiation time. Figure 1a shows the IRMPD mass spectrum of [Ubi+8 H]^8+^, which was obtained using 2 s irradiation at 3370 cm^−1^. Dominant products of dehydrated ubiquitin ions and fragment ions of ${y}_{18}^{3+}$ were observed, with other fragment ions, ${y}_{18}^{2+}$, ${y}_{24}^{3+}$, ${y}_{37}^{5+}$, ${y}_{44}^{5+}$, ${b}_{32}^{3+}$ and ${b}_{58}^{5+}$, also identified. Figure 1b shows the spectrum of [Ubi+10 H]^10+^, which was obtained using 4 s irradiation at the same wavelength. Spectra of other ions with different charge states from +7 to +13 were found to similar (Figs S1 and S2 in the Supporting Information). Generally, the fragment ions observed in these experiments are similar to those obtained with the traditional top-down methods^3^, such as CAD or IRMPD based on a CO_2_ laser^2^. As this absorption at 3370 cm^−1^ is mainly attributed to N-H vibrational modes of the ions, it is suggested that this IRMPD method might be used as an alternative top-down method for analyzing protein ions.

**Figure 2:**
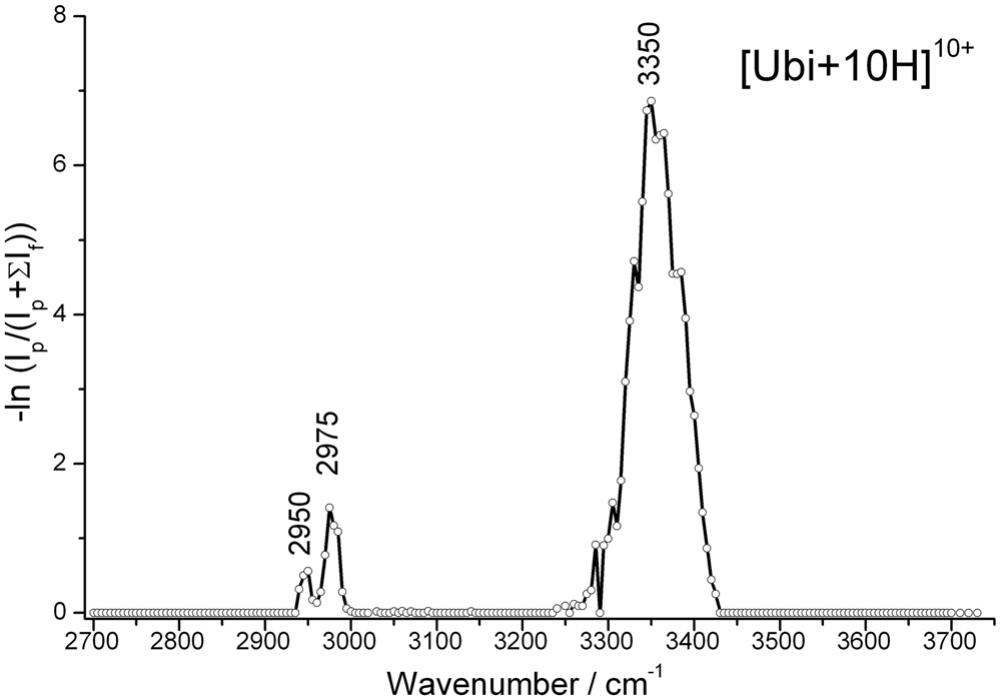
IRMPD spectrum of [Ubi+10 H]^10+^ in the range 2700–3740 cm^−1^.

By changing the output frequency of the OPO laser, and calculating the corresponding fragmentation efficiencies, the IRMPD spectra of ubiquitin ions were successfully measured. Figure 2 shows the spectrum of [Ubi+10 H]^10+^ in the region 2700–3740 cm^−1^. The spectrum is characterized by a dominant peak at 3350 cm^−1^, and two distinguishable weak peaks at 2950 cm^−1^ and 2975 cm^−1^. No absorption in the regions of 2700–2900 cm^−1^, 3050–3230 cm^−1^, or 3540–3740 cm^−1^ was found even with an extended irradiation time of 40 s. The positions of the absorption peaks are consistent with those in IR spectra of proteins obtained in the solution phase, but the line widths are much narrower. The full width at half maximum (FWHM) of the peak at 3350 cm^−1^ is about 70 cm^−1^, while the values for the 2950 cm^−1^ and 2975 cm^−1^ peaks are lower than 20 cm^−1^. Compared to the previously reported IR photodissociation spectra of the electron capture activated ubiquitin ions, [Ubi+7 H]^6+^ and [Ubi+8 H]^7+^, in the region 3050–3750 cm^−1^, the absorption peak at 3350 cm^−1^ is quite consistent, but the line width is much narrower^6^.

**Figure 3:**
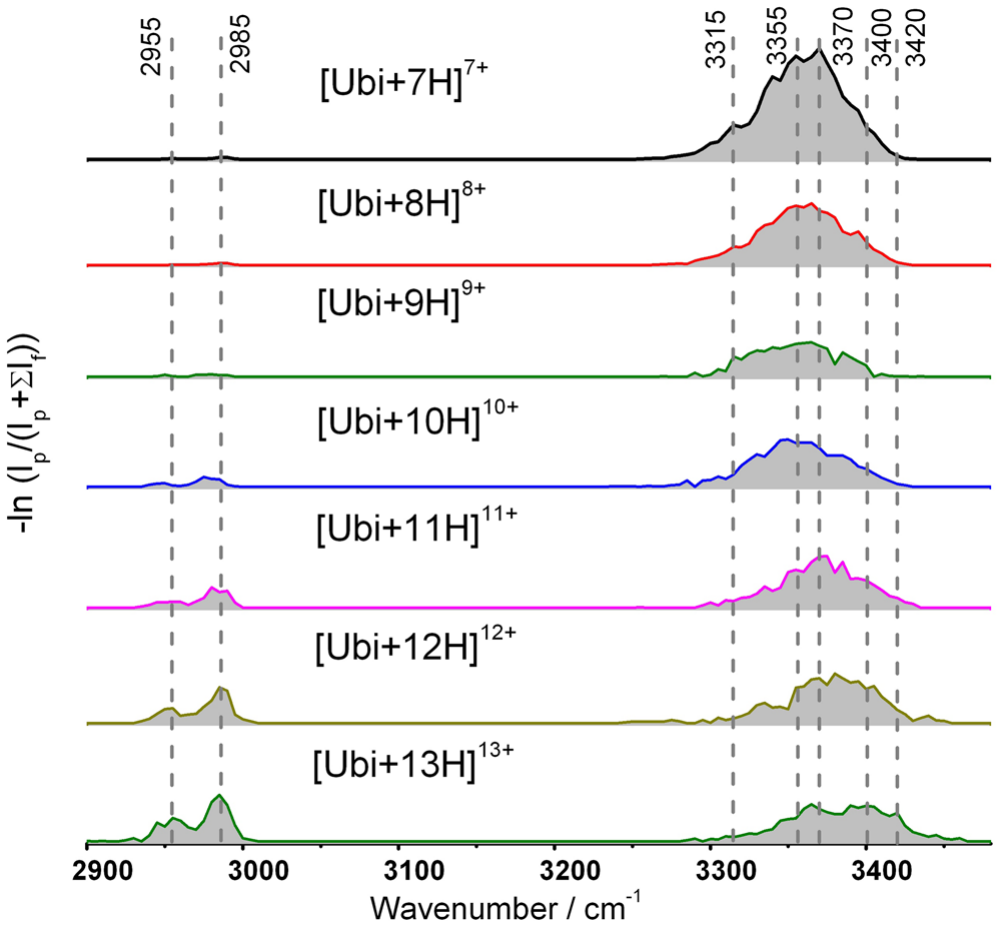
IRMPD spectra of [Ubi+nH]^n+^ (7 ≤ n ≤ 13) in the range 2900–3480 cm^−1^.

**Figure 4:**
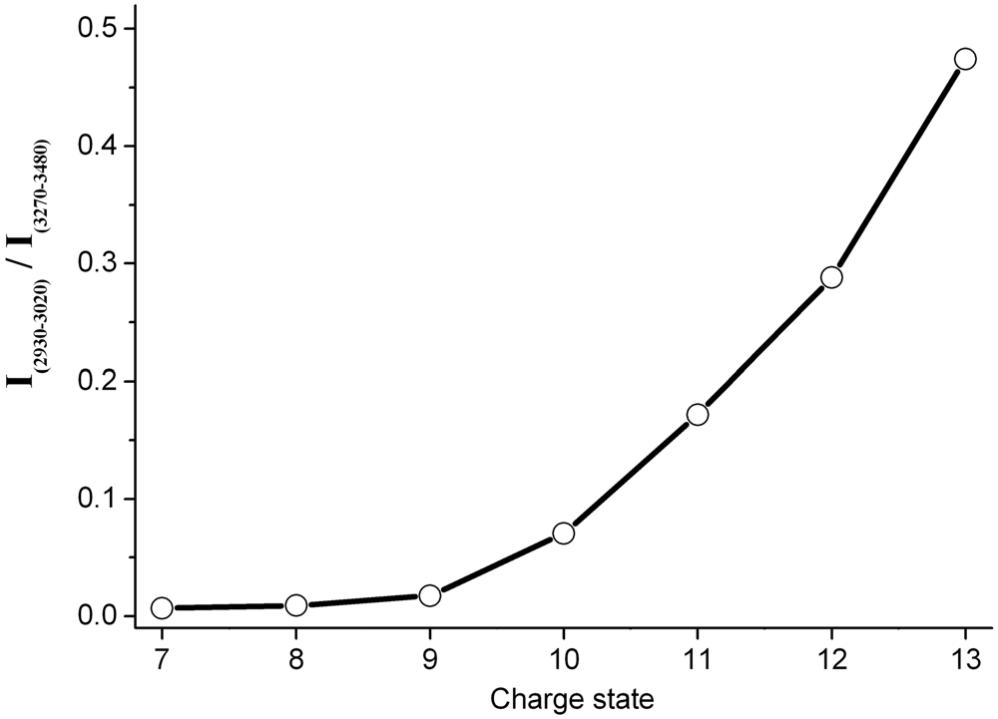
Comparison of ratios of integrated intensities in the ranges 2930–3020 cm^−1^ and 3270–3480 cm^−1^ for differently charged ions as a function of their charge states.

The primary question in the present study is whether the IRMPD spectra of the protein ions are distinguishable, raising the issue of the suitability of the method for revealing structural characteristics of gas-phase protein ions. Figure 3 shows the IRMPD spectra of [Ubi+nH]^n+^ (7 ≤ n ≤ 13) in the region 2900–3480 cm^−1^. Although similar absorption bands for all the ions can be observed, they are characterized by several differences. Firstly, while all ions show absorption near to 2985 cm^−1^, the intensities relative to the band at 3370 cm^−1^ vary among ions with different charge states. The ratios of the integrated IRMPD spectral intensities of the two regions were calculated, and are shown in Fig. 4. It is clear that the relative intensities of the 2985 cm^−1^ band for the ions, [Ubi+nH]^n+^ (7 ≤ n ≤ 9), are much weaker than those of [Ubi+nH]^n+^ (11 ≤ n ≤ 13), with the values increasing rapidly from 0.02 for [Ubi+9 H]^9+^ to 0.47 for [Ubi+13 H]^13+^. Secondly, for the ions [Ubi+nH]^n+^ (10 ≤ n ≤ 13), the 3370 cm^−1^ peak progressively broadens on the high-frequency side with increasing charge state. For example, all of the ions [Ubi+nH]^n+^ (7 ≤ n ≤ 10) show negligible absorptions at 3420 cm^−1^; however, for [Ubi+nH]^n+^ (11 ≤ n ≤ 13), the absorptions are quite strong, and increase with increasing charge state (Fig. 3).

The broadening of the peak at 3370 cm^−1^ with increasing charge state reflects that some O-H and/or N-H hydrogen bonds were weakened due to the increase in charge, causing some frequencies to be ~30 cm^−1^ less red-shifted than those observed in the cases of ions with low charge states. These changes are subtle for the low-charged ions, [Ubi+nH]^n+^ (7 ≤ n ≤ 10), but more obvious for the high-charged ions, [Ubi+nH]^n+^ (11 ≤ n ≤ 13). These results can be rationalized by the structural changes caused by the greatly increased Coulomb interactions due to the increase in the number of protons^6^. On one hand, the tertiary structures of gaseous protein ions weaken progressively with an increase in charge, which means that the protein ions change from a folded conformation to an unfolded conformation. An extreme example of this is the case of [Ubi+13 H]^13+^. Experimental results from ECD^6^ and ion mobility spectrometry^12^ have demonstrated that this ion has an extended conformation. On the other hand, González Flórez’s results showed the Coulomb-driven transitions in secondary structures from mostly helical to extended C5-type hydrogen-bonded structures^35^, which could also weaken the hydrogen bonds in secondary structures greatly.

**Figure 5:**
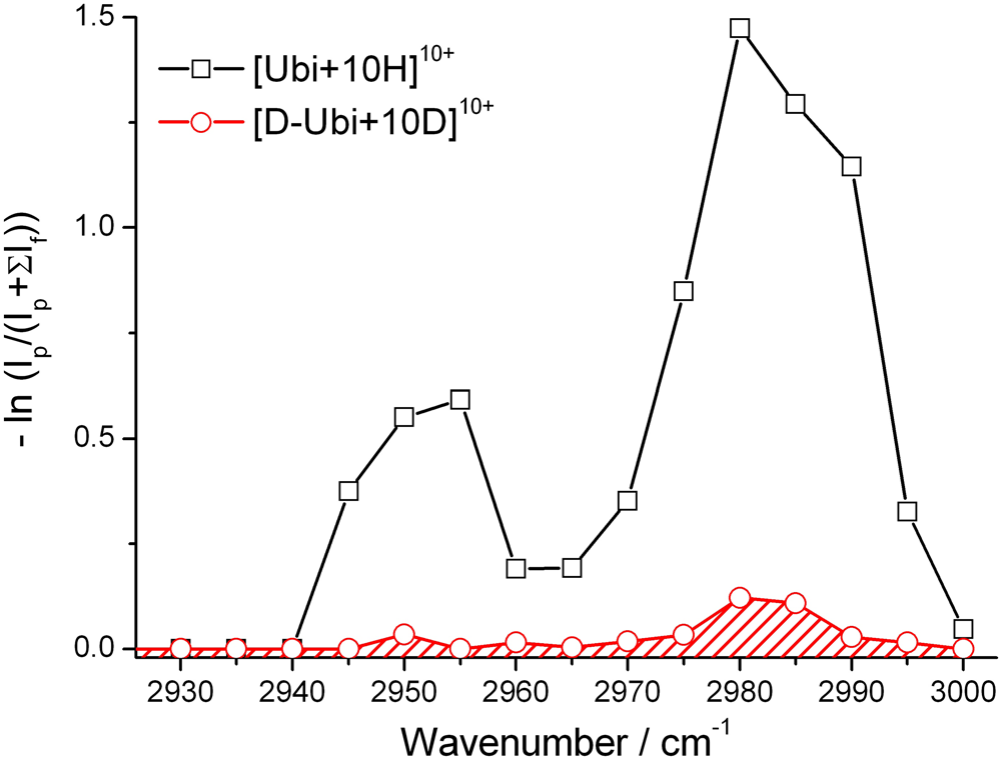
IRMPD spectra of ubiquitin ions and deuterated ubiquitin ions (both 10^+^) in the range 2930–3000 cm^−1^.

Generally, absorption in the range 2940–3000 cm^−1^ is also observed for proteins in the condensed phase. This band has been previously observed for the gaseous protein ions of cytochrome c and myoglobin^32,34^. It is usually interpreted as corresponding to C-H vibrational modes. However, these bonds are known to be stable, rarely hydrogen-bonded, and their vibrational intensities are lower than those of N-H or O-H. Thus, the relevance of charge state to the relative band intensity observed in Fig. 4 needs to be considered further. In order to better understand the origin of the observed absorption in the 2940–3000 cm^−1^ region, a sample of deuterated ubiquitin was prepared and studied. ESI MS showed that for the observed deuterated ubiquitin ions, more than 99% of all labile H atoms (including protons, and all H atoms in N-H and O-H) were replaced by deuterium atoms (Fig. S3 in Supporting Information). Interestingly, it was found that the absorption in the 2940–3000 cm^−1^ was also greatly decreased after the deuteration. Figure 5 shows the spectrum of [Ubi+10 H]^10+^ as an example. No significant shift was found for the peaks at either 2950 or 2980 cm^−1^, but their intensities were decreased to approximately 1/10 of those before the deuteration. For the deuterated ions of [Ubi+9 H]^9+^, [Ubi+11 H]^11+^ and [Ubi+12 H]^12+^, similar results were observed (Fig. S4 in Supporting Information).

Considering that almost all labile H atoms, including protons, H atoms in N-H and O-H, were replaced by deuterium atoms in these experiments, the weak absorption in the region 2940–3000 cm^−1^ after deuteration can be only interpreted to be C-H vibrational modes. However, it is unclear why their intensities decreased so much after the deuteration of the labile H atoms (Fig. 5). While the exact reason for this awaits to be further investigated, we briefly consider several possible explanations. The first suggestion is that the change of vibrational frequencies and mode-mode coupling after deuteration may modify the rate of intramolecular energy redistribution, consequently modifying the effective absorption cross-section and dissociation kinetics. The second suggestion is that the main contribution for the peaks in the region of 2940–3000 cm^−1^ for the undeuterated samples, in fact, is from hydrogen-bonded N-H and O-H groups, instead of C-H groups. The first suggestion is supported by some results previously reported by other research groups^39–41^. For example, Moritsugu *et al*. studied the mode coupling in myoglobin by the method of molecular dynamics simulation. They found that during the process of intramolecular vibrational energy transfer, the energy was transferred from a given normal mode to a very few number of selective modes, which were selected by their coupling coefficients^39^. This energy transfer characteristics can be applied here to explain why the deuteration can affect the mode-mode coupling relative to the C-H modes, since the highly coupled frequencies shifted after deuteration, reducing mode-mode coupling in the protein backbone. Besides, it is also confirmed that the coupling coefficients exhibited a correlation with the degree of the geometrical overlap between the coupled modes^39–41^. And this might to be applied here as an important factor to explain the intensity difference of the C-H vibrational mode for different charge states. For the second suggestion, it is agreed that the disruption of the tertiary structures due to Coulomb interactions induced by high charge states frees some hydrogen bonds that stabilize tertiary structures. Thus some new hydrogen bonds between the side chain N^+^–H/N-H groups and the amide carbonyl groups in the backbone might form and shift their vibration modes to the 2940–3000 cm^−1^ ^38^. However, this suggestion was rejected by the fact that the observed peaks at 2950 and 2980 cm^−1^ were characterized with very narrow line-widths. In other words, these bands appeared too narrow to be N-H’s or O-H’s involved in strong hydrogen bonds. Further investigations to better understand the detailed dynamics in such a system are still needed very much. Some experimental and theoretical methods applied in the study of energy flow in proteins^41–44^, might be expanded or reformed to apply for these protein ions in the gas phase.

In summary, we have obtained the direct IRMPD spectra of ubiquitin ions with the aid of a high energy OPO laser. The fragment ions produced though IRMPD are similar to those previously observed in CAD MS and IRMPD MS using a CO_2_ laser. Ubiquitin ions with different charge states showed absorption in the two regions of 2940–3000 cm^−1^ and 3280–3400 cm^−1^. A broadening of the absorption peak on the high-frequency side in the second region with increasing charge state reflects that some hydrogen bonds were weakened due to the increase of charge, which can be explained by the break of side-chain hydrogen bonds maintaining the tertiary structures of the protein ions and/or the unzipping of helixes caused by Coulomb interaction^37^. On the other hand, the relative peak intensity in the first region compared to that in the second region increased with increasing charge state. However, experiments also show that peaks in the region of 2940–3000 cm^−1^, which is assigned as C-H vibrational modes, decreased greatly after the deuteration of all labile hydrogen atoms. The most possible explanation for this is that the coupling coefficients between the C-H mode and other selective modes according to their frequencies decreased greatly after the deuteration, causing the energy distribution inside the protein ions and the consecutive absorption of IR photons less effective. Briefly, the direct IRMPD method reported here provides a technique for gas-phase protein ion structure study that might be complementary to present methods. We hope this work will prompt further experimental and theoretical efforts to understand the detailed dissociation and energy redistribution dynamics in the IRMPD of gas-phase protein ions.

## Experimental Method

Experiments were performed on a 7.0 T Fourier transform ion cyclotron resonance (FT ICR) mass spectrometer employing a Z-spray ESI source (IonSpec, Varian Inc., Lake Forest, CA, USA). Bovine ubiquitin was electrosprayed with an infusion rate of 120 µL/h to produce different charge state ions from solutions: 7+, 8+ in H_2_O/MeOH, 99:1; 9+−13+ in H_2_O/MeOH/MeCOOH, 49:49:2. Protein samples were obtained from Sigma–Aldrich, and used without further purification. The sample of deuterated ubiquitin was prepared by dissolving solid ubiquitin samples in D_2_O for 24 h, and a followed dilution with MeOD/D_2_O/MeCOOD (49:49:2) prior to the ESI experiments. All MS Spectra were recorded in the m/z range of 400–2000.

The precursor ions were isolated in the cell using the stored waveform inverse Fourier transform (SWIFT) method^45^. The experimental set up for IRMPD spectroscopy has been described previously^18^. Briefly, IRMPD spectra of selected ions were obtained by excitation of the O-H, N-H, and C-H stretching vibrations in the range 2700–3750 cm^−1^ by using an OPO laser (Firefly-IR, M Squared, UK). The typical laser was run in the broad linewidth mode, and the output power and linewidth were 600 mW and 7 cm^−1^, respectively. The irradiation time (with a typical value of 4 s) was controlled using a mechanical shutter (Sigma-Koki, Japan). The IR intensity was calculated by I = −ln (I_p_/(I_p_ + ΣI_f_)), in which I_p_ and I_f_ were the intensities of precursor and fragment ions, respectively. Considering the ICR detector response is proportional to the number of charges of the trapped ions, their peak intensity values were divided by their charge number^5^. All spectra were normalized with respect to the IR power.

## Electronic supplementary material

Supplemental Information for Charge-state Resolved Infrared Multiple Photon Dissociation (IRMPD) Spectroscopy of Ubiquitin Ions in the Gas Phase
