# Supplementary material for: Charge-state Resolved Infrared Multiple Photon Dissociation (IRMPD) Spectroscopy of Ubiquitin Ions in the Gas Phase
Source: Sci Rep. 2017 Nov 29;7:16592. doi: 10.1038/s41598-017-16831-2 (PMC5707388; doi:10.1038/s41598-017-16831-2)
Supplement: Supplementary file 1 — Supplemental Information for Charge-state Resolved Infrared Multiple Photon Dissociation (IRMPD) Spectroscopy of Ubiquitin Ions in the Gas Phase [file 41598_2017_16831_MOESM1_ESM.doc]

Supplemental Information for

**Charge-state Resolved Infrared Multiple Photon Dissociation (IRMPD) Spectroscopy of Ubiquitin Ions in the Gas Phase**

**Yijie Yang^1^, Guanhua Liao^1^, Xianglei Kong^1,2^***

1, State Key Laboratory and Institute of Elemento-Organic Chemistry, College of Chemistry, Nankai University, Tianjin, 300071 China
2, Collaborative Innovation Center of Chemical Science and Engineering, Nankai University, Tianjin 300071, China

Address reprint requests to Xianglei Kong, State Key Laboratory and Institute of Elemento-Organic Chemistry, Nankai University, Tianjin 300071, China. Tel.: 86-22-23509564, fax: 86-22-23502654, e-mail: [kongxianglei@nankai.edu.cn](mailto:kongxianglei@nankai.edu.cn)


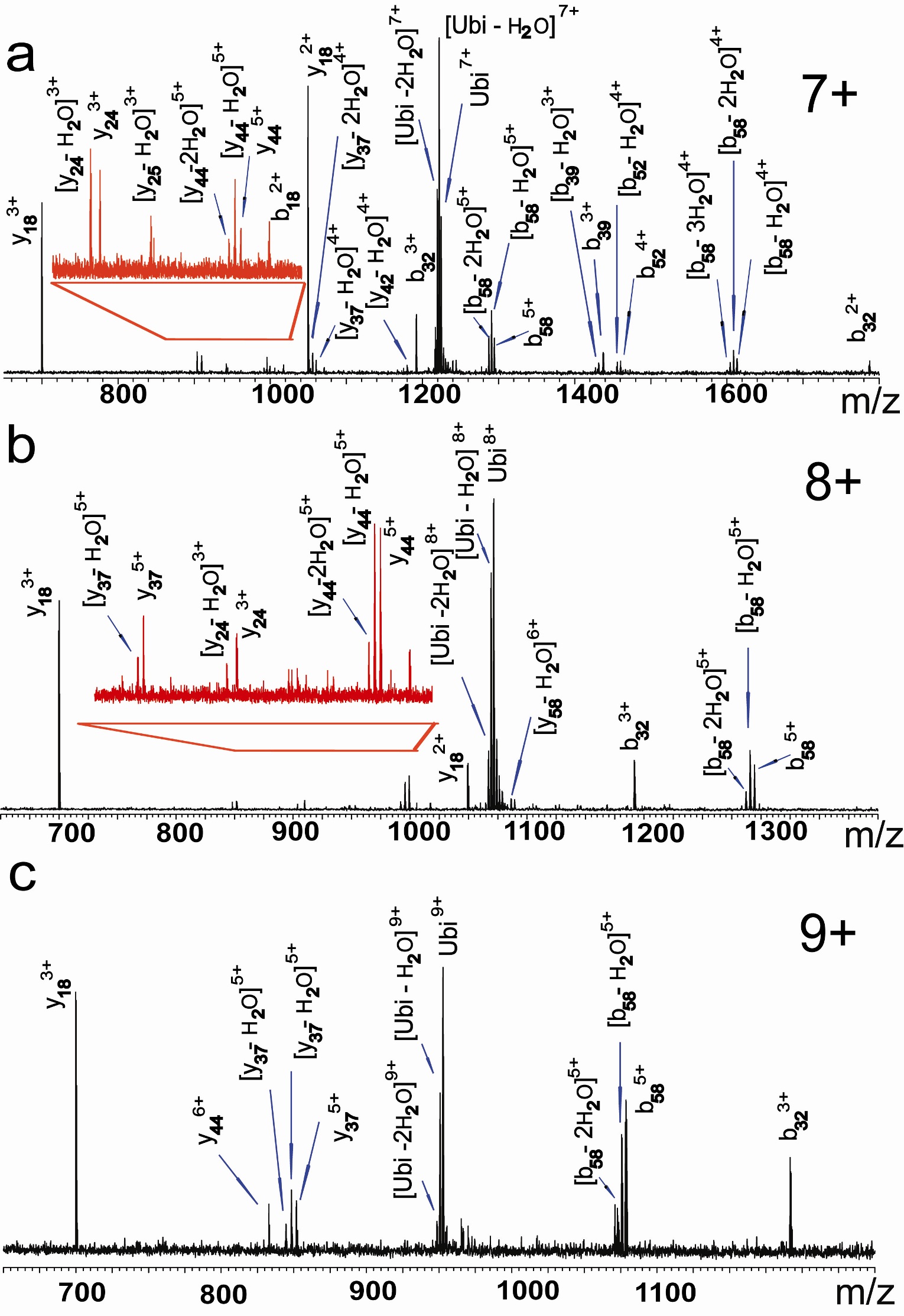


***Figure S1.*** IRMPD mass spectra of (a) [Ubi+7H]^7+^ , (b) [Ubi+8H]^8+^ and (c) [Ubi+9H]^9+^.


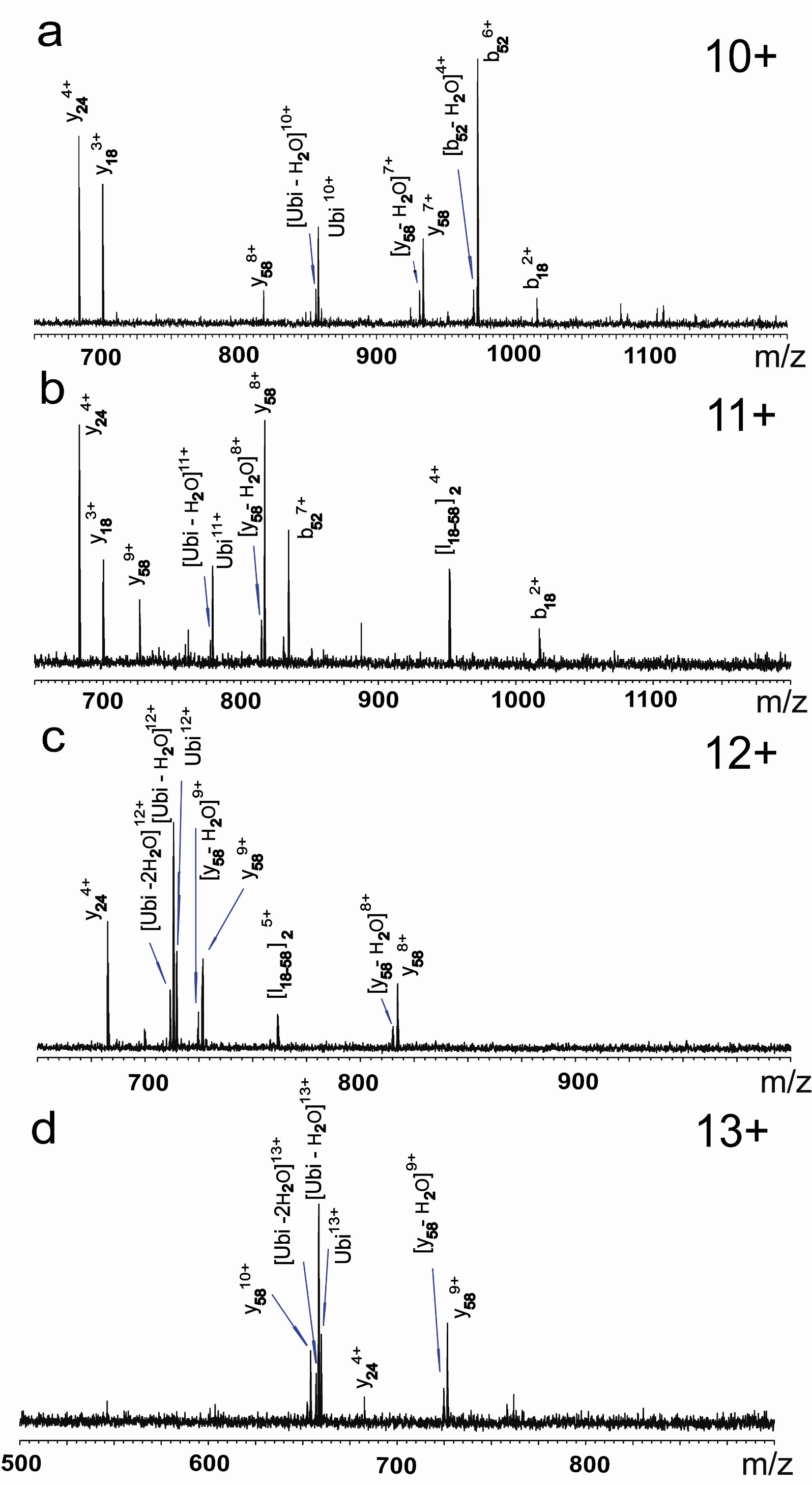


***Figure S2.*** IRMPD mass spectra of (a) [Ubi+10H]^10+^, (b) [Ubi+11H]^11+^, (c) [Ubi+12H]^12+^ and (d) [Ubi+13H]^13+^.


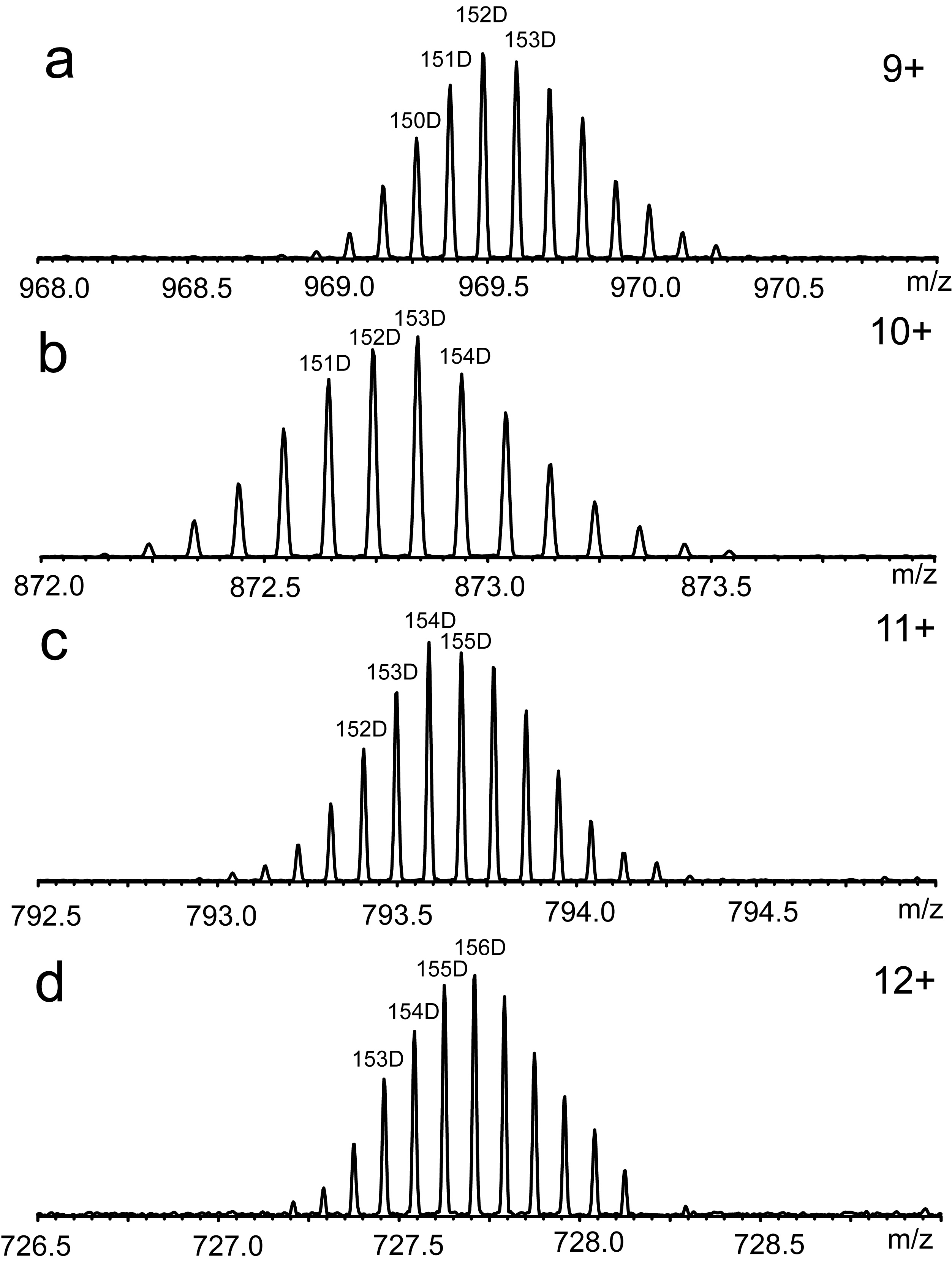


***Figure S3.*** Mass spectra of deuterated ubiquitin ions of (a) [D-Ubi+9D]^9+^, (b) [D-Ubi+10D]^10+^, (c) [D-Ubi+11D]^11+^ and (d) [D-Ubi+12D]^12+^. The narrow isotropic distributions of these ions peaked at the position of 152D-156D, indicating that almost all exchangeable H atoms were exchanged (the number of exchangeable H atoms in a neutral molecule is 144; considering the numbers of protons, the maximum numbers for 9+ - 10+ ions are 153-156).


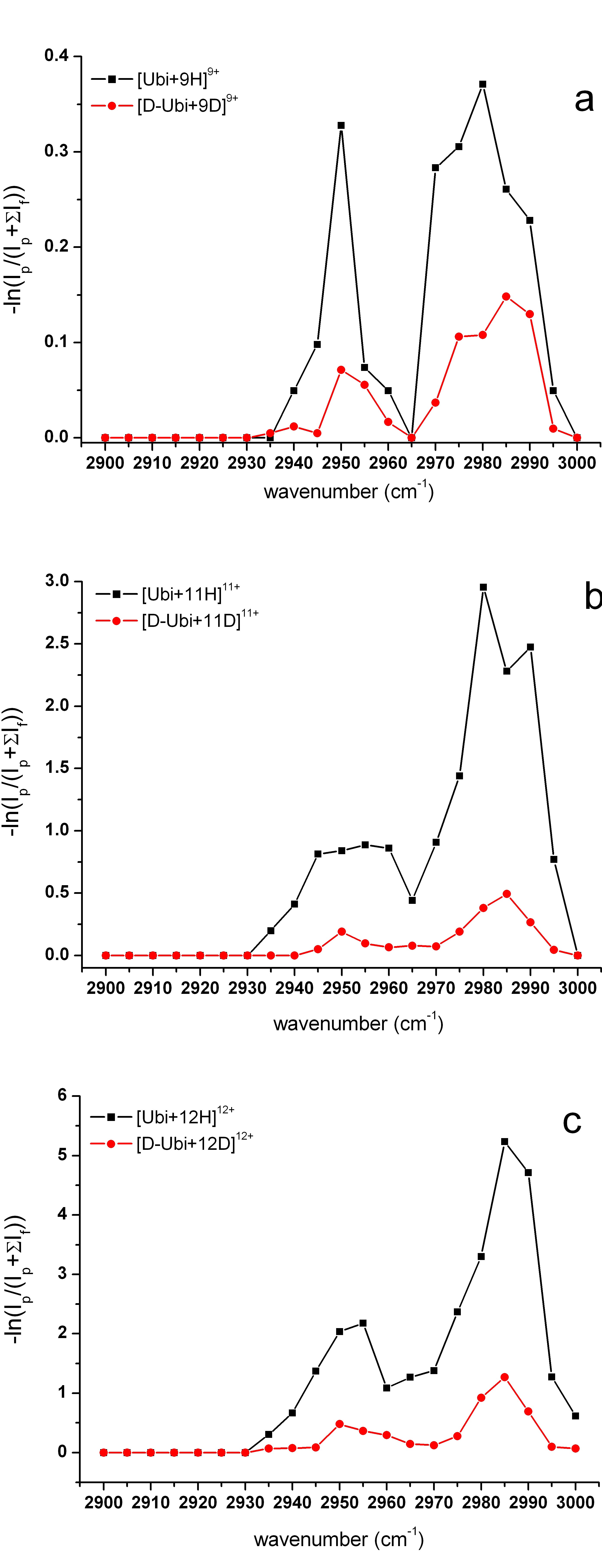


***Figure S4.*** IRMPD spectra of ubiquitin ions and deuterated ubiquitin ions a) 9+, b) 11+ and c)12+, in the range 2930–3000 cm^-1^.
